# Understanding participation psychology in rural cultural activities: internal and external drivers toward regional cultural sustainability

**DOI:** 10.3389/fpsyg.2025.1688408

**Published:** 2025-11-13

**Authors:** Changyun Zou, Yun Peng, Hong Han

**Affiliations:** School of Public Affairs and Administration, University of Electronic Science and Technology of China, Chengdu, China

**Keywords:** regional cultural, rural cultural activities, cultural sustainability, participation psychology, driving mechanisms

## Abstract

**Introduction:**

Cultural sustainability is recognized as the “fourth dimension” within the framework of sustainable development and has increasingly attracted attention from both academics and policymakers. Cultural activities serve as essential means for cultural expression and heritage preservation, playing a vital role in maintaining regional cultural sustainability. This study focuses on the psychology of participation in rural cultural activities, exploring both the internal and external driving mechanisms and action pathways to address the challenges of low participation rates and high demands for heritage preservation.

**Methods:**

This study takes Shatan Village in Guizhou, China as a case study, collects 158 valid samples through on-site questionnaire, uses SPSS and AMOS software for quantitative analysis, constructs a structural equation model (SEM) to examine the relationship between each variable and the moderating effect of government support.

**Results:**

The study found that the residents’ intention has a significant positive impact on actual participation behavior; cultural subjective norms have a significant positive effect on participation intention, while cultural attitudes and perceived behavioral control do not have significant effect on participation intention. Additionally, Government support acts as a moderating factor in the relationship between cultural attitudes, perceived behavioral control and participation intention at different levels, but has no significant moderating effect on the relationship between subjective norms and participation intention.

**Discussion:**

This study illustrates that regional cultural sustainability involves not only the protection of cultural resources but also the ongoing stimulation of residents’ internal psychological motivation. To effectively enhance enthusiasm for participation in cultural activities, it is essential to consider the interactions between individual cognition, social norms and institutional support. The research results provide a reference for shifting regional cultural inheritance from “passive acceptance” to “active creation” in different cultural contexts worldwide.

## 1 Introduction

Cultural sustainability, derived from the broader concept of sustainable development (Soini and Birkeland, 2014), has gradually emerged as a vital dimension complementing the social, economic, and environmental pillars of sustainability (Gerber, 2018). Since UNESCO officially integrated culture into the Sustainable Development Goals framework in 2015 (The UNESCO Courier, 2017), scholars and policymakers have increasingly emphasized culture’s role in fostering sustainable societies (Duxbury et al., 2016; Turner, 2017; Wiktor-Mach, 2020; Chen and Chen, 2025). Recent research has further highlighted that the realization of sustainability goals must account for cultural contextual diversity and local adaptation (Zheng et al., 2021; Ordonez-Ponce, 2023). Existing studies on cultural sustainability—rooted mainly in sociology, communication, and management (Soini and Birkeland, 2014; Baioni et al., 2021; Magliacani, 2023)—focus predominantly on heritage preservation (Hosagrahar et al., 2016) and cultural dissemination (Chen and Chen, 2025). However, static “museum-style” protection has proven inadequate to meet contemporary needs. Instead, the Sustainable Development Goals (SDGs) call for locally grounded, cross-disciplinary innovation (Moallemi et al., 2020), as sustainable development increasingly relies on active public participation and engagement (Macnaghten and Jacobs, 1997).

For example, scholars Lou and Xu (2023) have noted that the conventional museum’s “object-based” approach to communication lacks interactivity and public engagement. Transitioning to a “people-oriented” interactive model in a participatory context can improve the public’s sense of involvement and cultural identity. Similarly, creating an interactive mechanism that connects “space-culture-residents” within community cultural field can transform culture from a passive display into a vibrant community experience where everyone contributes to and enjoys culture (Zhou and Huang, 2019). This perspective emphasizes that cultural sustainability is not merely about the static preservation of cultural heritage; it is also a dynamic process that requires ongoing participation and interaction from the community. In this study, cultural sustainability is defined as the process of achieving the preservation of local culture and the reproduction of social value through the cognitive, emotional, and behavioral participation of community residents. This process is facilitated by continuous and inter-generational cultural practices.

Cultural activities are vital for cultural expression and inheritance (Tjarve and Zemīte, 2016; Sančanin, 2019) and play an increasing role in promoting cultural sustainability (Silva et al., 2023). For example, the annual Songkran Festival attracts tourists, enhances the local economy, and reinforces residents’ cultural identity while gaining international recognition (Minqi et al., 2024). Similarly, rural cultural activities sustain regional culture through interactions with tourism and commerce (Intason, 2021). However, rapid urbanization has caused an outflow of young people, leading to the decline of local customs and community rituals (Zhou, 2025). Without systematic mechanisms for protection and inheritance, symbolic forms of traditional heritage face the risk of disappearing (Newisar et al., 2024), threatening both cultural diversity and community cohesion. In this context, recent studies have highlighted that community awareness is a crucial psychological antecedent for participation in heritage preservation and cultural sustainability practices. Higher awareness enhances residents’ perceived value and sense of responsibility toward local culture, thereby motivating engagement in cultural activities (Umar et al., 2023; Çardak, 2025). Meanwhile, the active involvement of local authorities and heritage stakeholders, such as village leaders, curators, and community organizers, serves as a key enabling factor that strengthens trust, enhances institutional legitimacy, and increases the perceived meaning of participation (Tan et al., 2018; Calvi et al., 2022). These findings underscore that awareness cultivation and stakeholder engagement are essential external catalysts for sustaining cultural participation in rural contexts. Low participation in cultural activities, despite the urgent need for cultural inheritance, is shaped by both internal and external factors. Among these, cultural policies play a decisive role (Bonet et al., 2023). Participation in cultural activities is crucial for sustaining heritage and reproducing rural cultural capital (Chen, 2017), making policy support essential for promoting inheritance and rural development (Xu et al., 2019). Studies reveal that residents often seek stronger institutional backing for community engagement, as observed in the Xijiang Miao heritage site in southwest China (Hu et al., 2022). Yet, a paradox frequently arises: despite substantial government investment, community enthusiasm remains low (Zhang, 2024), weakening the outcomes of cultural initiatives and community cohesion.

This study focuses on participation in rural cultural activities, examining how rural residents engage in such activities and the internal and external mechanisms that shape their psychology of participation. Drawing on the Theory of Planned Behavior (TPB; Ajzen, 1991), this study deconstructs the internal drivers—attitude, subjective norms, and perceived behavioral control—that influence individuals’ participation intentions and behaviors. The TPB provides a robust theoretical lens for explaining how these psychological constructs determine behavioral intention and actual participation. However, the TPB alone offers limited insight into how external institutional or policy factors shape these psychological mechanisms. To address this gap, this study introduces “government support” as a moderating variable within the TPB framework, allowing us to investigate how external policy interventions interact with internal psychological processes to influence cultural participation in rural communities. By integrating internal and external determinants, this study contributes to advancing psychological understanding of cultural participation, extends the theoretical boundaries of TPB, and provides practical implications for fostering regional cultural sustainability. Specifically, the study pursues the following research objectives:

1. To examine the internal psychological factors (attitude, subjective norms, and perceived behavioral control) influencing rural residents’ intention and behavior in cultural participation.
2. To test the moderating role of government support on the relationship between internal drivers and participation intention.
3. To provide theoretical and practical insights into how psychological mechanisms can promote regional cultural sustainability through active community participation.

## 2 Theoretical development and hypothesis

### 2.1 Theoretical framework

The theoretical framework of this study is primarily based on the Theory of Planned Behavior (TPB) and the concept of cultural sustainability. It aims to explain the psychological mechanisms that drive individual participation in rural cultural activities and the pathways that lead to this participation. The TPB, developed by renowned psychology professor Icek Ajzen, is a social psychological model designed to predict and explain human behavior.

**Figure 1:**
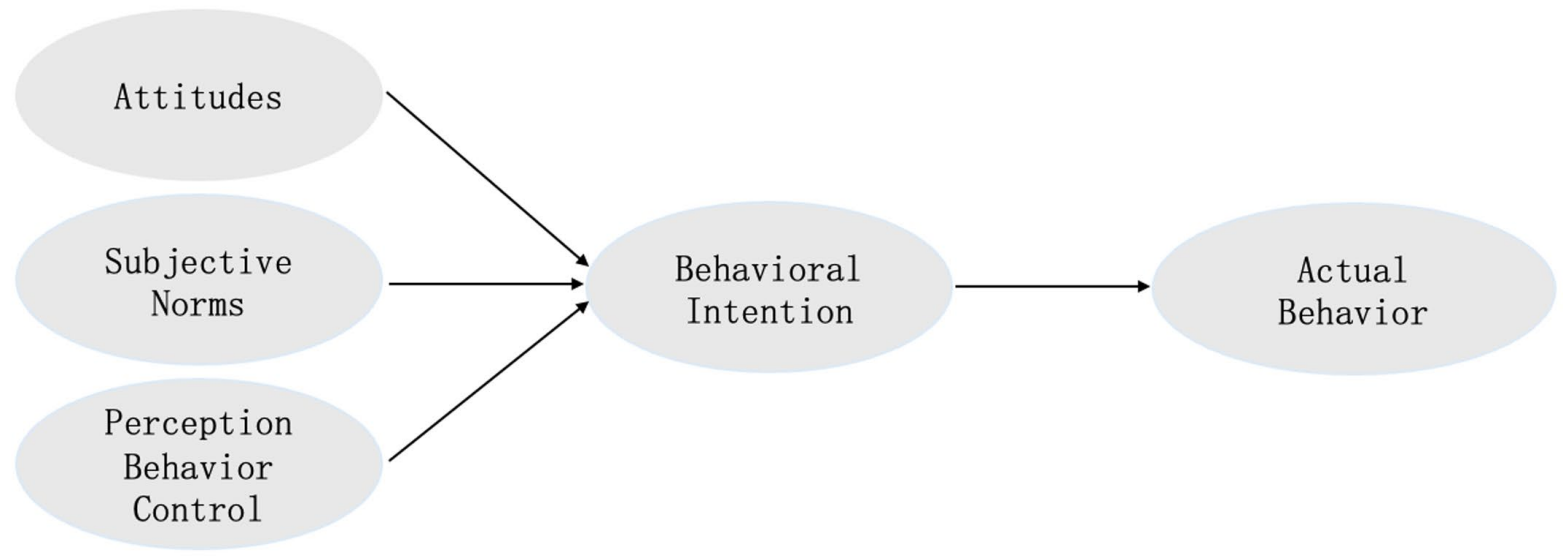
The TPB model adopted in this study.

According to this theory, whether an individual engages in a specific behavior is largely determined by their “behavioral intention,” and behavioral intention is jointly influenced by three factors, including behavioral attitude, subjective norms and perceived behavioral control. Behavioral attitude refers to an individual’s stable evaluation and preference for a specific object. Subjective norms which reflects the social pressure felt by individuals when making decisions, that is, the cognition of social expectations and norms. Perceived behavioral control refers to an individual’s perception of the difficulty required to complete a specific behavior (Ajzen, 1991). Based on the above conceptual description of the TPB framework, the basic theoretical model adopted in this study is illustrated in Figure 1.

In recent years, the TPB has been widely used in many fields such as psychology, management, and sociology, including e-commerce and consumer behavior (Hansen et al., 2004), environmental behavior (Chen, 2016), culture and tourism (Quintal et al., 2010) and other research topics. Importantly, many of these studies have confirmed the applicability of TPB in predicting participation in cultural events (Lin and Lee, 2020; Mo et al., 2023). For instance, research focused on heritage protection (Zhang and Ran, 2024), cultural tourism (Platania et al., 2021), and traditional art festivals (Shen, 2014) has shown that specific variables within the TPB framework significantly influence individuals’ willingness to engage in cultural heritage practices. In rural cultural settings, TPB helps disentangle whether residents act from inner valuation (attitude), social pressure (norm), or perceived ease (PBC), which is essential for policy design.

The academic research on the TPB in the field of culture and tourism can be divided into two levels. The first level assesses the effectiveness of TPB in explaining cultural participation behaviors. For example, studies have indicated that individuals’ attitudes toward cultural participation, social pressures, and perceived behavioral control significantly affect their willingness to participate and their actual behaviors (Ajzen and Driver, 1992). Analysis of cultural tourism behaviors has demonstrated a strong positive relationship between participation and factors such as personal attitude, subjective norms, and behavioral control (Lam and Hsu, 2006). The second level involves researchers introducing new variables that enhance the TPB framework. For example, studies have found that “self-efficacy” and “previous behavior” in sports activities are extended variables that have a significant impact on behavioral intentions and actual behaviors (Hagger et al., 2002). Other studies have integrated “perceived risk” and “perceived uncertainty” into the TPB model to investigate the tourism attitudes of tourists from various countries (Quintal et al., 2010). This expansion of the Theory of Planned Behavior allows for a more comprehensive understanding of participation behaviors.

### 2.2 Research hypotheses

According to relevant literature,within the framework of TPB, an individual’s behavioral intention is influenced by behavioral attitude, subjective norm, and perceived behavioral control. This study aims to explore how these factors affect rural residents’ intention to participate in cultural activities, and further consider the moderating role of government support.

#### 2.2.1 Activity participation intention and behavior predictors

Intention is often considered a key predictor of actual behavior (Montano and Kasprzyk, 2015). The test results of participation intention can help managers determine whether the event is worth further consideration (Peña-García et al., 2020). In the framework of the TPB, behavioral intention is a direct antecedent variable of actual behavior. In addition, community psychology research also shows that individuals’ intention to participate largely determines whether they will translate this intention into actual action (Chen et al., 2012), and this relationship is particularly prominent in collective action and community development.

When it comes to participation in traditional cultural festivals, it was also found that behavioral intention is the most important factor explaining directional behavior (Düşmezkalender et al., 2019). In the study of cultural heritage reuse, it is also shown that behavioral intention has a significant positive impact on residents’ actual behavior (Li et al., 2024). All these studies indicate that in the process of participating in cultural-related activities, individual behavioral intention is a crucial predictor of actual participation behavior. According to Ajzen (1991), behavioral intention reflects the extent to which individuals are inclined to engage in specific behaviors. In this study, we transformed it into the intention to participate in rural cultural activities. During our visits to areas where cultural events were held, we noted that a lack of intention to participate poses a significant barrier to cultural inheritance and development. Therefore, the first research hypothesis of this study examined the influence of the intention to participate in cultural activities on actual participation behavior.

H1: The intention to participate in cultural activities has a positive impact on actual participation behavior.

Ajzen (1991) pointed out that behavioral intention reflects an individual’s motivation and aim for a specific behavior, which is usually determined by behavioral attitude, subjective norms, and perceived behavioral control. Attitudes can often be influenced by desirable psychological motivations such as ‘trust’ (Lien and Cao, 2014), which means that attitudes may change over time as individuals re-evaluate something (Shaouf et al., 2016). Some research results in cultural contexts have also consistently shown that attitude is an important factor in determining individual psychological tendencies. For example, in a study on whether residents are willing to participate in the creation of tourist destinations, the attitudes of local community residents were significantly positively correlated with their behavioral intention to create cultural tourist destinations (Emmanuel, 2023). In a study on explaining residents’ support for the protection of local intangible cultural heritage, it was also shown that there was a significant positive correlation between residents’ attitudes and their behavioral intentions (Li et al., 2024). Thus, it is evident that positive cultural attitudes stem from the recognition of cultural heritage and community development, and the intention of rural residents to participate in cultural activities will increase. Based on this, the second research hypothesis is proposed.

H2: The cultural attitudes of rural residents have a significant positive impact on their intention to participate.

Individuals’ behavioral intentions are not only influenced by their personal attitudes, but also constrained by the social environment (Ajzen, 1991). Subjective norms represent the collective personal beliefs that others, related to the individual, think they should or should not engage in a specific behavior (Ajzen, 2005). In this study, cultural subjective norms refer to the support and expectations that rural residents feel from people in the same community. Generally, if the role models around them think they should act in a certain way, they will be more inclined to do so (Schepers and Wetzels, 2007). Studies on cultural tourism have also shown that subjective norms have a positive impact on the intention to repeatedly attend local festivals (Vesci and Botti, 2019). In the field of cultural tourism consumption, it is also proven that subjective norms have a positive impact on the consumption intention of tourists in intangible cultural heritage areas (Zhang et al., 2020). The literature indicates that if peers approve a behavior, individuals are more likely to engage in it. Therefore, we propose hypothesis 3.

H3: The cultural subjective norms of rural residents have a significant positive impact on their intention to participate.

In the framework of the TPB, perceived behavioral control is established as a determinant between intention and actual behavior (Ajzen, 2002). It is defined as an individual’s subjective judgment of whether individual is capable of performing a certain behavior. Therefore, in the context of participation in rural cultural activities, people who cannot control the situation may not be inclined to participate in it. For example, in the study of traditional village protection, it is shown that perceived behavioral control has a significant positive impact on their participation behavior (Liu et al., 2022). In the study of explaining the protection of intangible cultural heritage (Gejia batik), the perceived behavioral control of local residents was also positively correlated with their behavioral intention (Li et al., 2024). This indicates that if rural residents believe they possess sufficient resources and skills, their willingness to participate is likely to increase significantly. In the context of this study, factors such as time, resources, and skills may influence the perceived behavioral control of rural residents. Therefore, drawing on the support of the Theory of Planned Behavior (TPB) and related empirical research, we propose the following hypothesis.

H4: Rural residents' perceived cultural behavioral control has a significant positive impact on their intention to participate.

#### 2.2.2 The moderating role of government support

In the framework of the Theory of Planned Behavior (TPB), cultural attitudes, cultural subjective norms, and cultural perceived behavioral control are key internal factors in predicting individual behavioral intentions. However, the sustainable development of cultural industries cannot rely solely on internal cultural factors. External institutional forces—particularly government support—play an indispensable role in shaping individual and collective engagement. The government’s executive capacity is the other key to the development of cultural industries (Fan and Xue, 2018). Therefore, achieving regional cultural sustainability still requires external support from the government (Chen, 2014). Even the rise of Korean K-pop culture over recent decades has been closely linked to government intervention and industrial policy (Park, 2008). The government can significantly enhance residents’ intention to protect and inherit local culture through policy formulation, financial support, and infrastructure development (Zhao and Kang, 2009). Unlike internal psychological constructs, government support is an actionable and adjustable factor within policy systems, enabling it to amplify or suppress the effects of internal drivers on behavioral intention. While constructs such as cultural pride and collective memory also shape participation (Dubnewick et al., 2018; Žilič Fišer and Kožuh, 2019), these factors are more endogenous and less amenable to direct policy manipulation. Hence, government support provides a more practical and policy-relevant pathway for testing external moderation within the TPB framework.

In this study, government support is defined as the sum of various governmental actions promoting cultural heritage and rural development, encompassing policies, funding, technology, social capital, and relational networks. By providing resources and creating favorable institutional conditions, government support can strengthen the influence of rural residents’ cultural attitudes, subjective norms, and perceived behavioral control on their participation intentions. Therefore, it is conceptualized as a moderating variable linking internal psychological mechanisms with external institutional contexts. To provide a coherent analytical structure, this study first proposes a general hypothesis that government support moderates the relationships between internal drivers (attitude, subjective norm, and perceived behavioral control) and participation intention. Given that each of these internal drivers reflects distinct psychological processes within the TPB framework, we further develop three specific moderation hypotheses to capture their potentially differentiated effects. Thus, this paper proposes the following hypotheses:

H5: Government support has a moderating effect between cultural attitudes and intention to participate.

H6: Government support has a moderating effect between cultural subjective norms and intention to participate.

H7: Government support has a moderating effect between perceived behavioral control and intention to participate.

**Figure 2:**
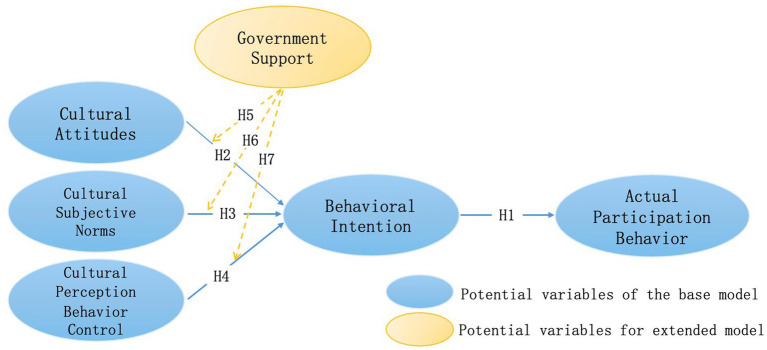
Research model.

In order to explore the influence mechanism between the internal factors (cultural attitudes, cultural subjective norms, perceived behavioral control) and behavioral intentions of rural residents, as well as the moderating role of external factors (government support) in this process, this study constructed a conceptual model as shown in Figure 2 based on all hypotheses.

## 3 Materials and methods

### 3.1 Sample and procedures

**Figure 3:**
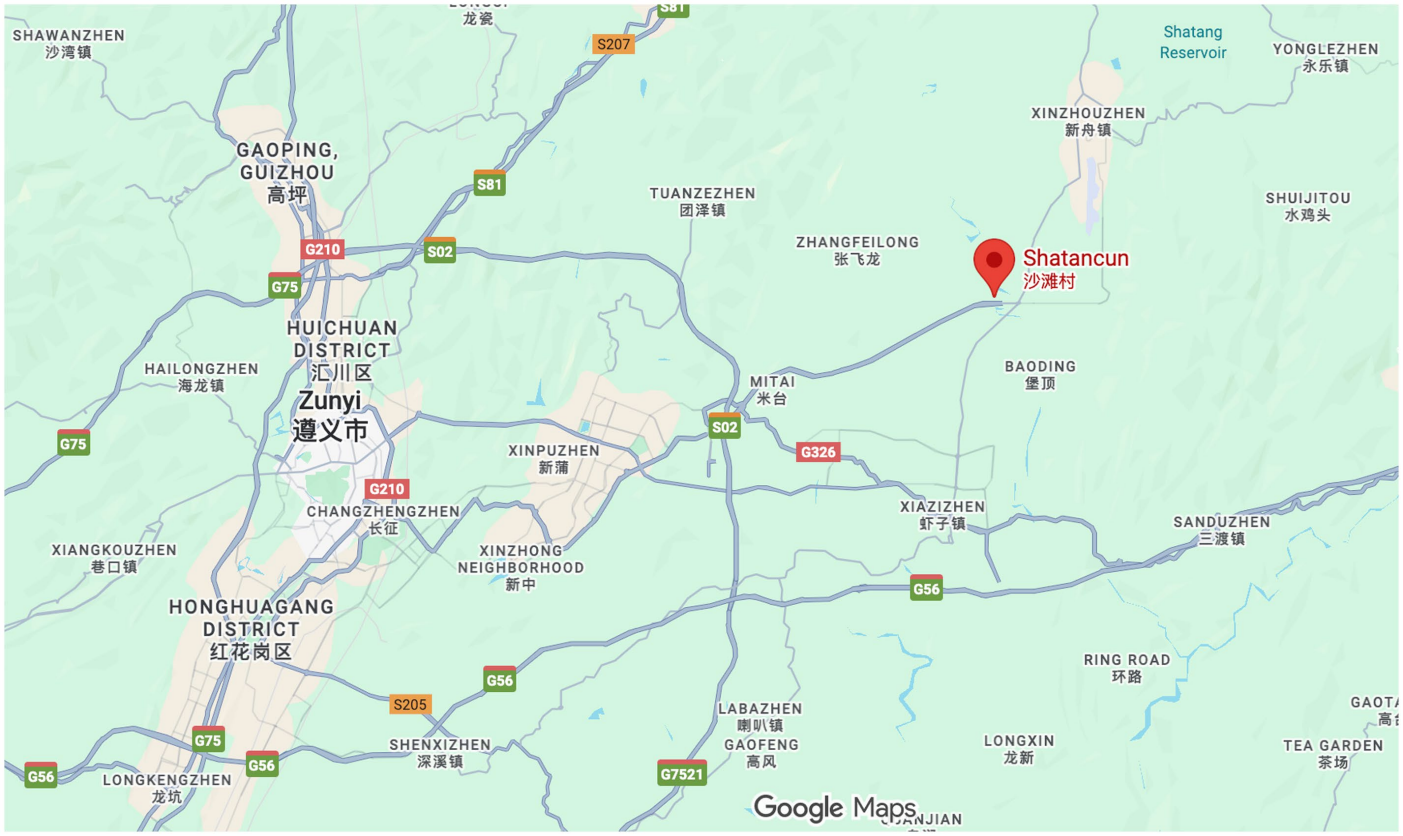
Map of the area surrounding Shatan village (screenshot obtained from Google Maps).

**Figure 4:**
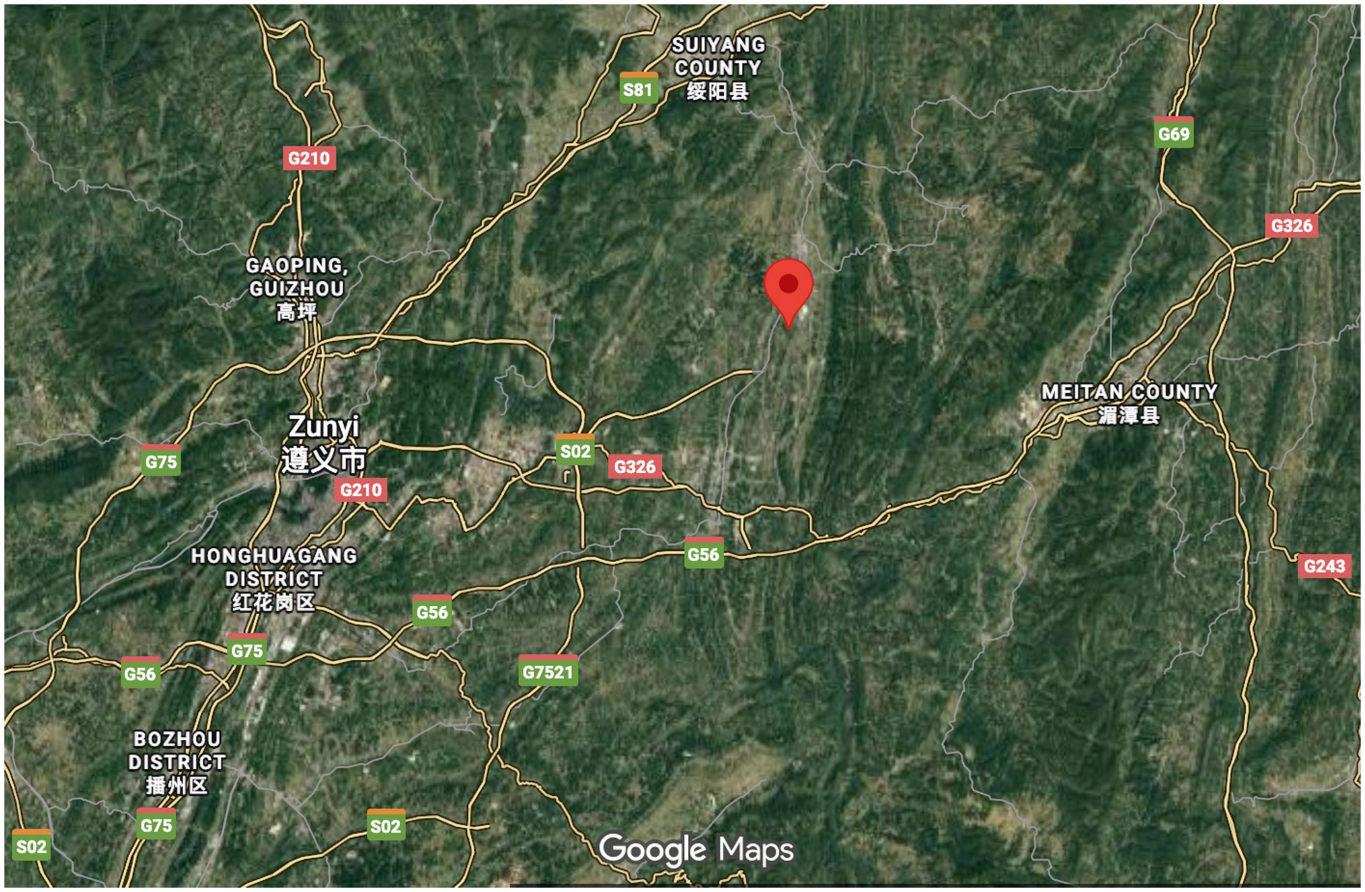
Topography surrounding Shatan village (screenshot obtained from Google Maps).

The sample data for this study is drawn from Shatan Village, located in the Xinpu New District of Zunyi City, Guizhou Province, China. Shatan Village is recognized as the birthplace of Shatan Culture (Wu, 2018) and covers an area of 8.8 square kilometers, with approximately 1,164 households. The map and topography surrounding Shatan Village are shown in Figures 3 and 4, which are screenshots obtained from Google Maps by entering “Shatan Village.” Shatan Culture is a regional cultural identity that developed in the mountainous region of northern Guizhou. Shatan culture is a regional culture that originated in the mountainous area of northern Guizhou. Over more than 300 years of development, it has gradually formed an academic and cultural tradition with Confucianism at its core. A local saying goes, “Guizhou culture is in northern Guizhou, and northern Guizhou culture is in Shatan.” This saying highlights the central importance of Shatan culture in the region. Shatan Village was selected as the focus of this research due to its rich cultural resources and its well-established rural revitalization projects.

To complete this study, we visited Shatan Village three times. The first visit took place in May 2024, covering five villages including Shatan and Shanbao Villages in Zunyi City. During this stage, we conducted in-depth interviews with local village leaders to gain a comprehensive understanding of the local cultural background and community structure. The second visit occurred in October 2024 and served as a pilot survey. During this stage, we found that some older villagers had limited educational backgrounds and faced difficulties in understanding certain questionnaire items. To improve the reliability and completeness of responses, informed consent was obtained before participation, and trained enumerators provided standardized verbal explanations when requested, following a unified guidance script to ensure understanding while maintaining response independence and confidentiality. Based on participants’ feedback and on-site observations, the questionnaire wording was refined to improve clarity and contextual appropriateness. The third visit, conducted in January 2025, marked the formal data collection stage. A total of 170 questionnaires were distributed, and 158 valid responses were obtained, yielding an effective response rate of 92.9%. The survey results showed that women accounted for 61.4% of participants, and men accounted for 38.6%. The majority of respondents were between 50 and 59 years old or above 60, representing more than 70% of the total sample. In terms of educational background, nearly 80% had a junior high school education or below. All respondents reported being familiar with “Shatan Culture.”

### 3.2 Measures

Our questionnaire utilized a mature scale that has been validated in previous studies to ensure content validity. During our second visit to Shatan Village, we reviewed the questionnaire to confirm that it was clear and easy to understand. The questionnaire comprised two sections. The first section focused on demographic variables, selecting gender, age, education level, and prior knowledge of “Shatan culture” as control variables. The second section encompassed the research variables, which included six measurement items: cultural attitude, cultural subjective norms, cultural perceived behavioral control, government support, behavioral intention, and actual behavior. Each of these measurement items contained three individual indicators aimed at evaluating the concept of regional cultural sustainability through rural cultural activities.

All concepts were assessed using a 5-point Likert scale, where respondents indicated their agreement ranging from 1 (strongly disagree) to 5 (strongly agree). The measurement for cultural attitude was adapted from the study by Lao et al. (Lao and Wang, 2015). Measurements for cultural subjective norms and cultural perceived behavioral control were based on the works of Li et al. (2024), Liu et al. (2022) and Li and Lu (2024). For behavioral intention and actual behavior, we referred to scales from Ajzen’s (2002) and Düşmezkalender et al.’s (2019) work. The measurement of government support was drawn from Kim et al. (2016) and Trang et al. (2016), complemented by three items designed based on the actual content learned during our field survey.

### 3.3 Statistical analysis method

The data analysis of this study adopted SPSS 21.0 and AMOS 24.0 software to process the original data. To ensure the accuracy and reliability of the analysis, we used SPSS 21.0 software for reliability analysis and AMOS 24.0 software for confirmatory factor analysis. In addition, we also used AMOS 24.0 software to analyze the model fit statistics to evaluate the measurement model. And construct a structural equation model (SEM) and moderation analysis to test the relationship between the variables. The quantitative analysis of the collected data in this study can reveal the relationship between the internal and external factors of rural residents’ participation in cultural activities. Overall, the questionnaire survey provided extensive quantitative data support, while the fieldwork supplemented the qualitative analysis through in-depth communication with rural residents and village leaders, thus providing background information and explanatory support for the discussion of quantitative data.

## 4 Results

### 4.1 Reliability and validity

The reliability and validity evaluation results of each latent variable in this study are shown in Table 1. The results of Cronbach’s alpha (*α*) and composite reliability (CR) of each construct exceeded the recognized threshold of 0.7, which indicates that each variable has good consistency within the construct and can stably measure the corresponding concept. In addition, the average variance extracted (AVE) of each variable is higher than the standard of 0.5, which also indicates that the construct has good convergent validity. Except for CA3, which is 0.674, the factor loading coefficients of each measurement item are also within the acceptable range in the case of overall measurement, and the others are all higher than the standard of 0.7, which further verifies the validity of the construct.

**Table 1:** Assessment of reliability and validity of variables.

Constructs	Items	α/CR	AVE	Factor loading
Cultural attitudes	CA1	0.747/0.754	0.506	0.748
	CA2			0.710
	CA3			0.674
Cultural subjective norms	CSN1	0.804/0.808	0.585	0.821
	CSN2			0.760
	CSN3			0.709
Cultural perception behavior control	CPBC1	0.875/0.884	0.719	0.788
	CPBC2			0.790
	CPBC3			0.954
Behavioral intention	BI1	0.860/0.863	0.678	0.887
	BI2			0.798
	BI3			0.780
Actual participation behavior	APB1	0.883/0.885	0.720	0.852
	APB2			0.793
	APB3			0.898
Government support	GS1	0.815/0.817	0.599	0.838
	GS2			0.709
	GS3			0.770

### 4.2 Correlation analysis and discriminant validity

Correlation analysis is a statistical method used to examine whether there is a relationship between variables and to assess the strength of that relationship. In this study, AMOS 24.0 was utilized to calculate the Pearson correlation coefficient for all dimensions. The results are presented in Table 2, indicating a significant positive correlation among the variables. In addition, this study compared the square root of the average variance extracted (AVE) of each construct with the correlation coefficient between the constructs referring to the Fornell-Larcker standard. According to Fornell and Larcker (1981), when the square root of the AVE of each construct is greater than the correlation coefficient between the constructs and any other constructs, it can be determined that sufficient discriminant validity has been obtained. The analysis results show that the AVE square root of all constructs in this study is greater than the correlation coefficient between the constructs, which indicates that each constructs effectively captures a unique concept. Overall, this study achieved good discriminant validity.

**Table 2:** Discriminant validity: Pearson correlation and AVE square root values.

	Cultural attitudes	Cultural subjective norms	Cultural perception behavior control	Behavioral intention	Actual participation behavior	Government support
Cultural attitudes	**0.711**					
Cultural subjective norms	0.216	**0.765**				
Cultural perception behavior control	0.194	0.264	**0.848**			
Behavioral intention	0.158	0.210	0.195	**0.823**		
Actual participation behavior	0.322	0.378	0.226	0.219	**0.849**	
Government support	0.326	0.287	0.332	0.359	0.287	**0.774**

The bold numbers are the square root values of AVE.

### 4.3 Model fit evaluation

Before the research hypothesis test, the overall model fit test of the structural equation model constructed in this study was first performed. The SEM model fit evaluation in this study selected chi-square value (χ^2^), degrees of freedom (df), chi-square freedom ratio (χ^2^/df) and multiple relative fit indexes as evaluation indicators as shown in Table 3. The results showed that CMIN/DF was 1.408, less than 3,while GFI, NFI, NNFI, and CFI all reached the standard of more than 0.9, and RMSEA was 0.051, less than 0.1. The values of each indicator met the evaluation criteria, the overall model fit was good, and the hypothesis model construction was supported.

**Table 3:** Model-fitting test results.

Fit index	Standard value	Statistics value
df	–	83
χ^2^	–	116.884
χ^2^/*df*	<3	1.408
GFI	>0.9	0.911
NFI	>0.9	0.901
NNFI	>0.9	0.960
CFI	>0.9	0.968
RMR	<0.08	0.128
RMSEA	<0.10	0.051

### 4.4 Testing hypothesis results

#### 4.4.1 Testing the relationship between cultural attitude, cultural subjective norm, cultural perceived behavioral control and intention to participate and actual participation behavior

Regression analysis was performed on research hypotheses 1, 2, 3, and 4, and the results are summarized in Table 4. According to the regression coefficient summary results, intention to participate has a significant positive impact on actual participation behavior (*β* = 0.254, *p* < 0.05), and hypothesis H1 is established. Cultural attitude has no significant effect on intention to participate in cultural activities (*β* = 0.142, *p* > 0.05), and hypothesis H2 is not established; cultural subjective norms have a significant positive impact on intention to participate in cultural activities (*β* = 0.234, *p* < 0.05), and hypothesis H3 is established; cultural perceived behavioral control has no significant effect on intention to participate in cultural activities (*β* = 0.153, *p* > 0.05), and hypothesis H4 is not established.

**Table 4:** Model regression coefficient summary table.

*X*	→	*Y*	Non-standardized coefficient	SE	*C*	*p*	Standardization coefficient
Behavioral intention	→	Actual participation behavior	0.254	0.089	2.862	0.004	0.256
Cultural attitudes	→	Behavioral intention	0.142	0.125	1.143	0.253	0.114
Cultural subjective norms	→	Behavioral intention	0.234	0.112	2.094	0.036	0.208
Cultural perception behavior control	→	Behavioral intention	0.153	0.111	1.385	0.166	0.124

Interestingly, the absence of significance for both cultural attitude and perceived behavioral control diverges from the classical TPB expectation. This pattern implies that, in the rural cultural context, participation intentions are not primarily driven by personal evaluations or perceived efficacy, but rather by collective expectations and normative pressures within tightly knit communities. In such environments, a “psychological–behavioral gap” may emerge when individuals’ positive cultural attitudes fail to translate into action due to institutional constraints or limited perceived agency. These findings indicate that subjective norms, rather than individual cognition, function as the dominant motivational force shaping participation in cultural activities within rural sociocultural structures. These new findings will be further elaborated with detailed interpretive commentary in the “Discussion of Research Findings” section, taking into account the specific context.

#### 4.4.2 Test of the moderating effect of government support

The moderating effect test was conducted with cultural attitudes, subjective cultural norms, and perceived behavioral control as independent variables, while behavioral intention served as the dependent variable and government support acted as the moderating. In order to reduce the impact of collinearity and maintain the explanatory power of the model, the independent variables and moderating variables were all mean-centered, and the dependent variable was not mean-centered. The processing is shown in Table 5, and the test results are shown in Table 6, Tables 7, and 8 respectively.

**Table 5:** Research variable processing description.

Type	Name	Data type	Data processing
Dependent variable	Behavioral intention	Quantitative	No processing
Independent variable	Cultural attitudes	Quantitative	Mean-centered
	Cultural subjective norms		
	Cultural perception behavior control		
Moderator variable	Government support	Quantitative	Mean-centered

**Table 6:** Results of the analysis of the moderating effect of government support on cultural attitudes.

	Model 1	Model 2	Model 3
Constant	3.774** (49.613)	3.774** (52.376)	3.816** (51.333)
Cultural attitudes	0.193* (1.994)	0.056 (0.576)	0.001 (0.007)
Government support		0.372** (4.343)	0.354** (4.154)
Cultural attitudes * Government support			−0.183* (−2.008)
Sample size	158	158	158
*R* ^2^	0.025	0.131	0.153
Adjusted *R*^2^	0.019	0.119	0.136

*p < 0.05, **p < 0.01. The *t* value is in brackets.

**Table 7:** Results of the analysis of the moderating effect of government support on cultural subjective norms.

	Model 1	Model 2	Model 3
Constant	3.774** (50.106)	3.774** (52.697)	3.803* (51.825)
Cultural subjective norms	0.223** (2.678)	0.124 (1.497)	0.058 (0.633)
Government support		0.352** (4.189)	0.346** (4.138)
Cultural subjective norms * Government support			−0.125 (−1.639)
Sample size	158	158	158
*R* ^2^	0.044	0.141	0.156
Adjusted *R*^2^	0.038	0.130	0.139

*p < 0.05, **p < 0.01. The *t* value is in brackets.

**Table 8:** Results of the analysis of the moderating effect of government support on cultural subjective norms.

	Model 1	Model 2	Model 3
Constant	3.774** (49.953)	3.774** (52.517)	3.833** (51.557)
Cultural perception behavior control	0.201* (2.486)	0.088 (1.082)	0.044 (0.533)
Government support		0.357** (4.174)	0.325** (3.812)
Cultural perception behavior control * Government support			−0.215* (−2.547)
Sample size	158	158	158
*R* ^2^	0.038	0.135	0.170
Adjusted *R*^2^	0.032	0.124	0.154

*p < 0.05, **p < 0.01. The *t* value is in brackets.

The moderating effect test of this study is divided into three models. Model 1 includes independent variables. Model 2 adds moderating variables on the basis of Model 1, and Model 3 adds the product term of independent variables and moderating variables on the basis of Model 2. From Table 6 presented above, it is clear that the interaction term between cultural attitude and government support is significant (*t* = −2.008, *p* = 0.046 < 0.05). This means that when cultural attitude affects participation intention, the influence range is significantly different when government support is at different levels, and hypothesis H5 is established; from Table 7 it is clear that the interaction term between cultural subjective norms and government support is not significant (*t* = −1.639, *p* = 0.103 > 0.05).

**Figure 5:**
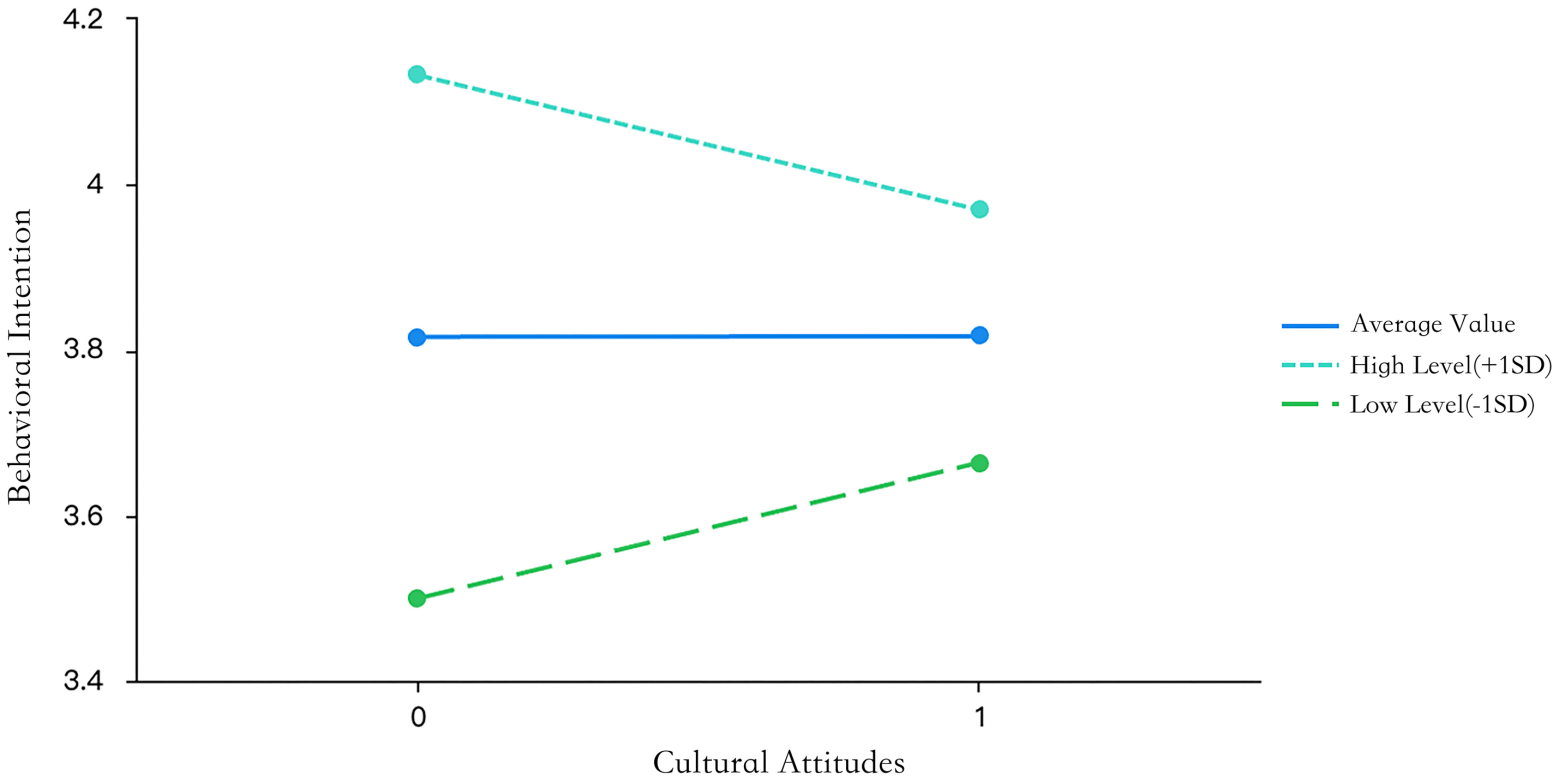
Results of cultural attitude effects.

**Figure 6:**
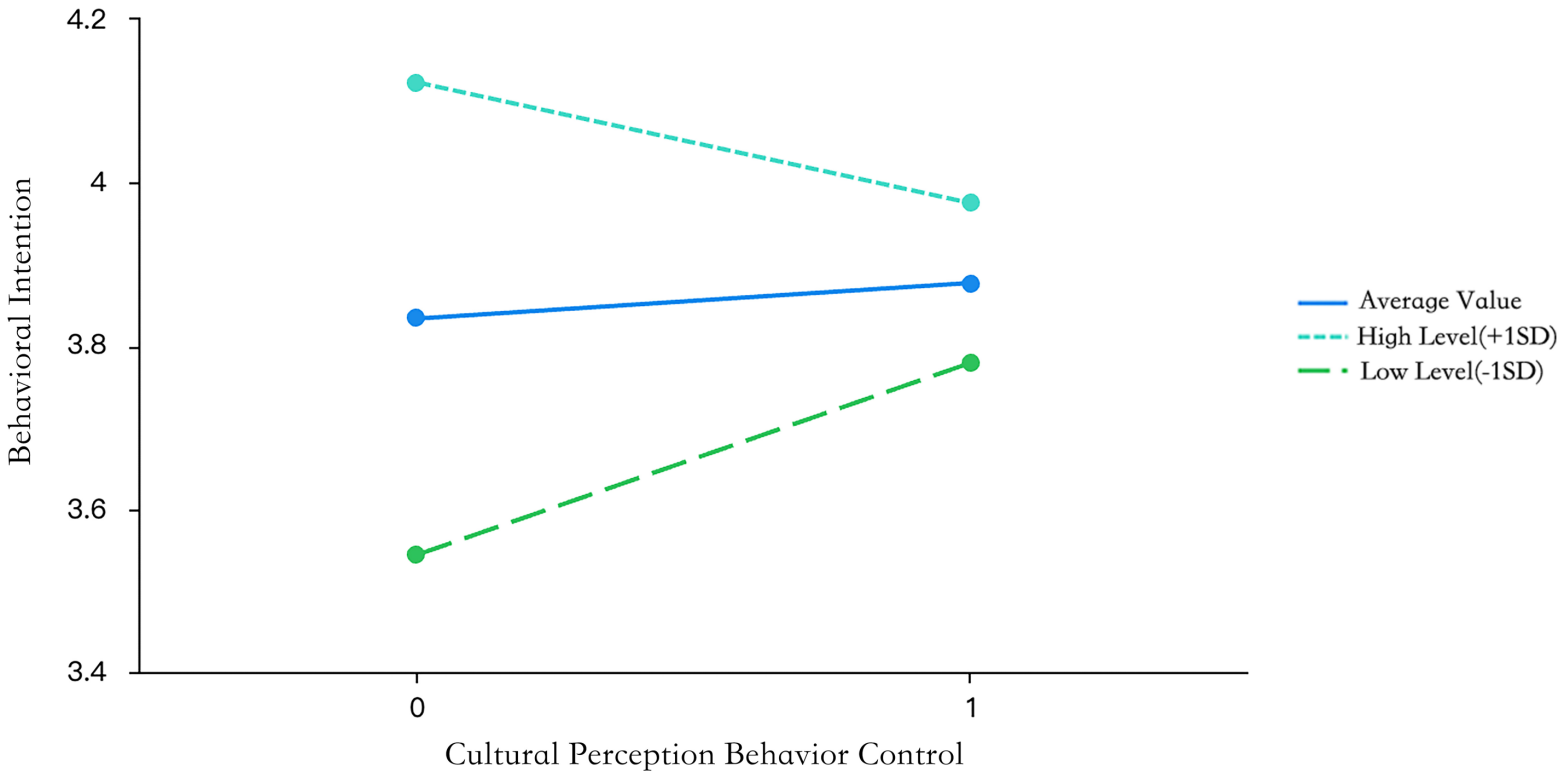
Results of cultural perception behavior control effects.

Meanwhile, it was shown in Model 1 that X has an influence on Y, which means that when cultural subjective norms affect participation intention, the influence range is consistent when government support is at different levels, and hypothesis H6 is not established. From Table 8, it is clear that the interaction term between cultural perceived behavioral control and government support is significant (*t* = −2.547, *p* = 0.012 < 0.05). This means that when cultural perception behavior control affects participation intention, the magnitude of the impact is significantly different when government support is at different levels, and hypothesis H7 is established. The specific moderating effect can be seen from the slope diagram (Figure 5, Figure 6). At different moderating levels, the direction and intensity of the effects of cultural attitudes and cultural perception behavior control on participation intention have changed. When the level of government support is high, the positive impact on participation intention is weakened; when the level of government support is low, the positive impact on participation intention is relatively stronger.

## 5 Discussion and conclusion

### 5.1 Discussion of research findings

#### 5.1.1 Internal driving factors of activity participation psychology

Based on the perspective of the TPB and the assumption that government support has a moderating effect, this study proposes the TPB expansion model to explore the influencing factors of rural residents’ participation in cultural activities. While supporting the basic theoretical framework of TPB, this study also reveals that under the specific regional cultural background and the characteristics of the local population, the action paths between the variables of this theory may be different, which needs to be discussed in depth in combination with the context.

First, this study further verified the fundamental premise of TPB that “behavioral intention is the direct antecedent of actual behavior” (Ajzen, 1991). Consistent with prior research in cultural festivals (Düşmezkalender et al., 2019) and cultural heritage reuse (Li et al., 2024), our findings confirmed a significant positive relationship between participation intention and actual behavior. Importantly, this study extends TPB by demonstrating that in rural cultural contexts, where residents often have lower education levels and live in relatively closed cultural environments, strong emotional and value-based connections to “regional culture” can reinforce the translation of intention into action. Behavioral psychology emphasizes that actual behavior reflects the integration of intention, self-coordination, cooperation, planning, self-consciousness, and personality (Lin et al., 2014). Our results suggest that even when practical obstacles exist, rural residents’ cultural participation is strongly driven by psychological intention, highlighting the importance of considering contextual and affective factors in TPB applications. This implies that for promoting rural cultural participation, the key is to stimulate and strengthen residents’ psychological intentions rather than relying solely on external mobilization, thereby extending TPB by emphasizing the role of culturally embedded motivations in bridging intention and behavior.

Secondly, cultural attitude did not significantly predict the intention to participate in cultural activities, which contrasts with the TPB proposition that attitude is a key determinant of intention (Ajzen, 1991) and differs from findings in cultural tourism and heritage conservation (Emmanuel, 2023; Li et al., 2024). This divergence may reflect contextual factors unique to rural settings. First, from a motivational perspective, attitudes alone may fail to generate intrinsic motivation or perceived benefits, weakening their influence on behavioral intentions (Ryan and Deci, 2000). Rural residents, as stakeholders in cultural sustainability, are affected by the interdependence of cultural resources, which may limit the translation of positive attitudes into actual participation (Wondirad et al., 2020; Li et al., 2024). Second, qualitative observations revealed that villagers often considered cultural activities “meaningful” but viewed them as the responsibility of younger residents or village leaders, who could gain social status through engagement. This illustrates an “attitude-behavior gap” in rural cultural participation, where positive attitudes do not necessarily lead to behavioral intentions (Bechler et al., 2021). These findings extend TPB by highlighting the moderating role of contextual, motivational, and social factors in shaping the attitude-intention link, suggesting that in rural cultural contexts, attitude alone may be insufficient to predict intention without considering local incentives and social norms.

In contrast, cultural subjective norms significantly and positively predicted participation intention in cultural activities, consistent with TPB expectations and prior studies on intangible cultural heritage and traditional festivals (Vesci and Botti, 2019; Zhang et al., 2020). This indicates that in rural communities shaped by collectivist culture, individual behavioral intentions are strongly influenced by social expectations. According to Hofstede’s cultural dimensionality theory (Hofstede, 2011), in collectivist contexts, individuals are more likely to conform to group norms to maintain social acceptance and identity (Choi and Geistfeld, 2004). In rural community systems, strong interpersonal ties and limited channels for public opinion dissemination mean that the attitudes of key figures, such as village leaders or family members, can easily solidify into group norms, forming an effective behavioral mobilization mechanism. These findings extend TPB by demonstrating how collectivist cultural contexts amplify the influence of subjective norms, suggesting that promoting rural cultural participation may be more effective through fostering a positive community cultural atmosphere than through external policy advocacy alone.

This study also found that cultural perception-behavior control had no significant effect on the intention to participate in cultural activities. This result deviates from the prediction path proposed in the TPB model and is different from Xia et al. (2020) and Li et al.’s work (2024) in the study of intangible cultural heritage participation. This article try to explain this phenomenon from the following perspectives: From a psychological perspective, on the one hand, it may reflect the lack of “self-efficacy” in rural society. According Bandura’s self-efficacy theory (Bandura, 1986), perceived behavioral control can only significantly affect an individual’s psychological motivation when the individual has a clear goal and a clear path. During our research and visits, we also found that many villagers did not regard cultural activities as something they “must participate” in. Villagers were unsure whether participating in cultural activities would bring actual benefits. This uncertainty would affect their sense of self-efficacy, limiting the incentive effect of a sense of behavioral control on their intention to participate. On the other hand, it is also possible that cultural activities in rural areas have not established sufficient participation mechanisms, and it is more likely due to concerns about institutional factors such as “complexity of activities,” “organizational opacity,” and “unclear processes.” This “perception of structural barriers” weakens the effectiveness of perceived behavioral control in TPB. These findings suggest that, in rural cultural contexts, the influence of perceived behavioral control on intention may be contingent upon both motivational and structural factors, offering a nuanced perspective on TPB. Practically, enhancing villagers’ cultural participation self-efficacy and providing clear, accessible participation pathways through training, rewards, and role modeling may help bridge this gap.

In summary, subjective norms play a significant role in explaining the intention to participate in cultural activities, while cultural attitudes and perceived behavioral control fail in specific contexts, suggesting that we need to go beyond a single psychological variable and combine social structure and cultural context for more explanatory theoretical expansion.

#### 5.1.2 External regulatory factors of activity participation psychology

The results of this study also verified that government support has a significant regulatory effect on the influence of cultural attitudes and cultural perception behavior control on the intention to participate in cultural activities.

First, government support appears to moderate the relationship between cultural attitudes and participation intention. The results indicate that high levels of government support weaken the impact of cultural attitudes on participation intention. This may seem counterintuitive (Zhao and Kang, 2009), as conventional wisdom suggests that greater government support should enhance positive attitudes. However, when external support is abundant, villagers may rely more on incentives provided by the government rather than internal cultural motivation, reflecting an “over-justification effect” (Pretty and Seligman, 1984). That is, excessive external reinforcement can lead individuals to attribute their behavior to external rewards rather than intrinsic motivation, thereby diminishing the role of internal attitudes. In this context, rural residents may perceive participation in cultural activities as a government-driven initiative rather than an expression of personal cultural identity, subtly altering the attitude–intention link and offering an insightful nuance to TPB applications in settings with strong institutional support.

However, government support did not play a moderating role between cultural subjective norms and intention to participate in cultural activities, which shows that in rural society, the constraints of family and collective norms are higher than government intervention. This also explains that in hypothesis 3, the willingness of rural residents to participate in cultural activities mainly depends on the behavior and expectations of the people around them, so the “external drive” of government support has limited moderating effect on this relationship. There are often strong “group norms” in rural society (Bai and Qi, 2022),” and the “energy” brought by such norms far exceeds the influence of external support. Therefore, although the government provides external support, in terms of participation in cultural activities, villagers are more inclined to act according to internal norms and community expectations. In addition, this phenomenon also reflects the shaping effect of social identity on individual behavior, that is, when an individual identifies with a social group, it is easier to follow the expectations of the group and less sensitive to external intervention.

Finally, government support moderates the relationship between perceived behavioral control and participation intention, consistent with Hypothesis 5. Interestingly, low levels of government support appear to strengthen the effect of perceived behavioral control on intention. From the perspective of identity theory (Stets and Serpe, 2013; Fang et al., 2023), when institutional guidance is limited, villagers actively engage in cultural activities to construct and reinforce their self-identity. In such cases, self-initiated participation enhances the motivating effect of perceived control. In contrast, higher levels of government support may reduce residents’ autonomy, as they rely more on external arrangements, which can dampen the influence of perceived behavioral control. This also explains the results presented in Hypothesis 5 and Hypothesis 7. These observations imply that in rural cultural contexts, the interplay between institutional support and individual identity considerations may condition the effectiveness of perceived behavioral control, providing a context-sensitive refinement to TPB predictions.

### 5.2 Conclusion

This study grounded in the Theory of Planned Behavior (TPB), constructs an integrated model of internal and external driving mechanisms to systematically examine the psychological processes and path relationships underlying rural residents’ participation in cultural activities. Unlike previous studies that primarily focused on internal cognitive factors or relied on qualitative analyses, this research extends the TPB framework by introducing institutional support (government backing) as a contextual moderating variable, thereby enriching the theoretical explanation of TPB within the domains of rural cultural participation and cultural sustainability. The main conclusions are as follows:

First, this study reaffirms the classical TPB proposition that behavioral intention directly predicts actual behavior, and verifies its applicability in rural cultural contexts. Individual willingness remains a key link in transforming intention into action, suggesting that policies promoting rural cultural participation should emphasize intrinsic motivation rather than external compulsion (Ajzen, 1991).

Second, among internal drivers, cultural subjective norms significantly influence participation intention, highlighting the guiding power of collective norms in rural settings. By contrast, cultural attitudes and perceived behavioral control show weaker effects, indicating that institutional constraints and low self-efficacy may hinder the translation of positive attitudes into actual intentions. This finding extends TPB by showing that psychological pathways are context-dependent.

Third, government support demonstrates a complex moderating role: while it can complement internal drivers, excessive intervention may crowd out intrinsic motivation, reducing self-driven participation. Its non-significant moderation on the “subjective norm–intention” path further suggests that community-based networks exert a more stable influence than top-down mechanisms. Practically, this implies that policy design should prioritize enabling rather than substitutive support to balance institutional guidance with local autonomy.

Overall, these findings refine the TPB framework by integrating institutional dynamics into the analysis of rural cultural participation, deepening our understanding of how psychological and contextual factors jointly shape cultural sustainability.

#### 5.2.1 Research implications

In the context of global cultural diversity facing risks of marginalization and disappearance, enhancing individuals’ enthusiasm to participate in local cultural practices has become a crucial path toward achieving regional cultural sustainability. The stimulation of participation psychology requires a comprehensive understanding of how internal cognition, social norms, and institutional support interact dynamically. Cultural sustainability is not only about resource protection but also about the continuous mobilization of psychological momentum.

First, more concrete incentive mechanisms should be designed to transform cultural attitudes into intrinsic motivation for long-term engagement. Local governments can introduce recognition-based incentives (e.g., “Cultural Contribution Awards” or symbolic honors) and micro-grant programs that support grassroots cultural events, encouraging residents to see participation as both socially valued and personally rewarding.

Second, local community organizations play an irreplaceable role in cultural inheritance. Village-level committees, family networks, and cultural volunteer groups should be empowered to establish community co-creation models—for example, co-designed cultural festivals, participatory heritage documentation, and peer-led workshops. Through mechanisms such as social reinforcement and role modeling (Bandura, 1963), these collective practices can strengthen individuals’ self-efficacy and shared responsibility in sustaining local culture.

Third, government support should be transformed from a directive to a facilitative role. Rather than substituting for community initiative, governments should provide structural guarantees (policy consultation, training platforms, small-scale financial matching) that enable autonomous community action and mitigate the “external motivation crowding-out effect.”

Finally, a psychological empowerment and motivation framework should be constructed to foster collective emotional connections and cultural identity. By integrating empowerment training with emotional engagement—such as storytelling projects or intergenerational mentorship—cultural inheritance can shift from “passive acceptance” to “active creation.”

This multidimensional participation model, combining incentive alignment, community collaboration, and policy facilitation, offers practical insights not only for rural China but also for community-based cultural governance worldwide, promoting sustainable and participatory cultural development across diverse contexts.

### 5.3 Limitations and future directions

Although this study provides valuable theoretical and empirical insights, several limitations should be acknowledged. First, the sample is regionally concentrated. Shatan Village in China was selected as the research site, which, despite its strong representativeness, may limit the external validity of the findings. Future research could adopt cross-regional or cross-cultural comparisons to examine whether these mechanisms hold under different sociocultural contexts. Second, as the study relies on cross-sectional data, it cannot capture the temporal evolution of participation psychology. Longitudinal or experimental designs would allow for a more dynamic understanding of how residents’ cultural attitudes, subjective norms, and perceived behavioral control evolve over time. Third, certain methodological challenges should be noted. Due to literacy constraints among some older participants, trained field assistants provided standardized verbal explanations when necessary, which may have introduced minor interviewer effects. Additionally, the use of self-reported data could lead to social desirability bias. Future studies might adopt mixed methods, such as interviews, focus groups, or visualized questionnaires, to enhance data validity and inclusivity. Finally, this study primarily examined three internal factors and one external moderator within the TPB framework. However, additional psychological constructs, including cultural pride, collective memory, and intergenerational identity, should be incorporated in future models to enrich theoretical comprehensiveness. Moreover, awareness and negative perception, as highlighted by previous studies, may play a crucial yet underexplored role in shaping participation psychology. Cultural awareness enhances individuals’ cognitive understanding and emotional engagement in cultural practices, whereas negative perceptions, such as perceived irrelevance, fatigue, or institutional distrust, may suppress participation intentions. Future research should therefore integrate these elements to develop a more nuanced and balanced understanding of how both positive and negative psychological forces influence cultural participation and sustainability. In summary, future research should deepen exploration of the psychology of cultural participation through multi-regional, longitudinal, and mixed-method approaches, while incorporating broader emotional and cognitive dimensions to provide stronger theoretical and empirical foundations for regional cultural sustainability.

## Data Availability

The original contributions presented in the study are included in the article/supplementary material, further inquiries can be directed to the corresponding author/s.
